# Macroinvertebrate Habitat Dynamics under Frequent Hydropower-Induced Discharge Fluctuations: Patch-Scale Metrics to Quantify Effects of Hydropeaking and Morphological Complexity

**DOI:** 10.1007/s00267-026-02574-2

**Published:** 2026-08-18

**Authors:** Aude Lecrivain, Giovanni De Cesare, Christine Weber, Nico Bätz

**Affiliations:** 1 Eawag, Swiss Federal Institute of Aquatic Science and Technology, Surface Waters—Research and Management, Kastanienbaum, Switzerland; 2 EPFL, Hydraulic constructions platform, Station 18, Lausanne, Switzerland

## Abstract

Hydropeaking, driven by intermittent hydropower production, induces frequent and rapid sub-daily discharge fluctuations that alter riverine habitats and cause biodiversity loss worldwide. Benthic macroinvertebrates are particularly vulnerable due to their limited mobility, complex life cycles, and sensitivity to rapid hydraulic change. Yet, tools to quantify hydropeaking impacts on macroinvertebrate habitats at ecologically relevant spatio-temporal scales remain limited. Here, we present and evaluate a patch-scale approach to quantify macroinvertebrate habitat dynamics under hydropeaking. Using high-resolution hydrodynamic modelling (0.5 m spatial, 10 min temporal resolution) in a 1 km alpine river reach, we generated a habitat time-series and derived four complementary metrics: habitat probability, habitat shifts, drift risk, and desiccation risk. We analyzed metric interactions, applied multimetric clustering to identify recurring habitat–risk regimes, and related these to local morphology-driven structural complexity. Results show that hydropeaking increases habitat dynamics, with frequent habitat shifts occurring even in patches with highest habitat probabilities. Structurally complex areas generally support higher habitat probability and lower drift risk, but may remain exposed to desiccation, revealing trade-offs among key hydraulic stress mechanisms. These patterns demonstrate that the metrics capture ecologically relevant habitat responses, while confirming that structural complexity alone cannot eliminate the stress induced by frequent hydropeaking. Overall, the proposed metrics provide an ecologically meaningful framework to study macroinvertebrate habitat dynamics under hydropeaking. By explicitly linking hydraulic stress mechanisms, habitat dynamics, and morphological context, the framework supports process-based river management in regulated rivers.

## Introduction

Benthic macroinvertebrates are one of the most diverse freshwater organism groups, including aquatic insects, snails, worms, and mussels, and are widely used as biological indicators of habitat quality and river health (He et al. 2024). Macroinvertebrates play key roles in nutrient cycling, organic matter decomposition, and energy transfer to higher trophic levels (Macadam and Stockan 2015). Understanding how discharge regulation alters macroinvertebrate habitats is therefore critical for river restoration and sustainable hydropower management, particularly in reconciling renewable energy production with biodiversity conservation.

Macroinvertebrate communities show a patchy distribution (Beisel et al. 2000; Béjar et al. 2020), reflecting the heterogeneity of conditions that form the habitat mosaic of a river reach (White et al. 2019). The interplay between the structural complexity of a channel (morphology) and the temporal variability in discharge (flow regime) generates a dynamic, shifting habitat mosaic (Stanford et al. 2005), within which local hydraulic stress varies among patches (Bätz et al. 2023, 2025) and shapes the composition of the macroinvertebrate communities (Friese et al. 2025). A patch can be defined as a geographically fixed area corresponding to the spatial and temporal perception of macroinvertebrates (Bätz et al. 2023), typically about 0.25–0.50 m² (Beisel et al. 2000; Béjar et al. 2020). Some patches serve as low-stress refugia, while others experience the full intensity of hydraulic change (Weber et al. 2013). Macroinvertebrates have evolved their life cycles and adapted to natural discharge pulses by using habitats and their dynamics for survival, dispersal and recolonization (Lake 2000).

However, human-induced discharge regulation increasingly disrupts natural dynamics, posing challenges for both biodiversity conservation and river management (He et al. 2024). Discharge regulation and hydropower production have altered flow regimes globally (Grill et al. 2019). Hydropeaking, the rapid and frequent variation in discharge caused by intermittent hydropower production, is one of the most disruptive forms of regulation (Bipa et al. 2024; Bozeman et al. 2025). As flexible hydropower gains importance in balancing renewable energy supply, hydropeaking frequency is expected to increase in the coming decade (Koolen et al. 2023) as will the associated ecological pressures. This is despite growing recognition of ecological hydropower mitigation, which is increasingly formalized through legally binding regulations.

Although adapted to natural discharge variability, macroinvertebrates are highly sensitive to discharge alteration (Bruno et al. 2010; Judes et al. 2023). Frequent hydropeaking increases habitat dynamics, triggers passive drift during rapid velocity increases (Naman et al. 2016; Tonolla 2023) and exposes patches to repeated drying, leading to stranding and egg desiccation (Miller et al. 2020; Jaulin Gautronneau et al. 2026). Macroinvertebrate response is therefore driven by processes operating at the patch scale, with hydropeaking amplifying habitat dynamics far beyond natural levels (Bätz et al. 2025). The strength and extent of these patch-scale stressors also depend on the local morphology and associated structural complexity, which are often simplified through human intervention (Wohl 2016).

Despite their sensitivity to hydropeaking, macroinvertebrates remain underrepresented in existing assessment frameworks (Hayes et al. 2023; Friese et al. 2025), which have largely focused on fish (Hayes et al. 2023; Bipa et al. 2024; Bozeman et al. 2025) or describing habitats in static rather than dynamic terms (Holzapfel et al. 2017; Ruhi et al. 2018). No approaches yet exist that link hydropeaking-induced habitat dynamics to the hydraulic stress mechanisms governing macroinvertebrate distribution at the patch scale. Their limited mobility relative to rapid hydraulic changes induced by hydropeaking, together with high community variability among rivers, challenges the development of broadly applicable approaches. To address this gap, this study builds on the concept of patch-scale habitat dynamics introduced by Bätz et al. (2023, 2025) and introduces a set of process-based metrics that explicitly quantify macroinvertebrate-relevant habitat dynamics under frequent hydropeaking. Specifically, we aim: (1) to define patch-scale metrics that quantify macroinvertebrate habitat dynamics in response to hydropeaking, and (2) to examine how these metrics interact to jointly describe habitat dynamics and how these interactions vary with morphology-driven structural complexity.

## Methods

**Fig. 1:**
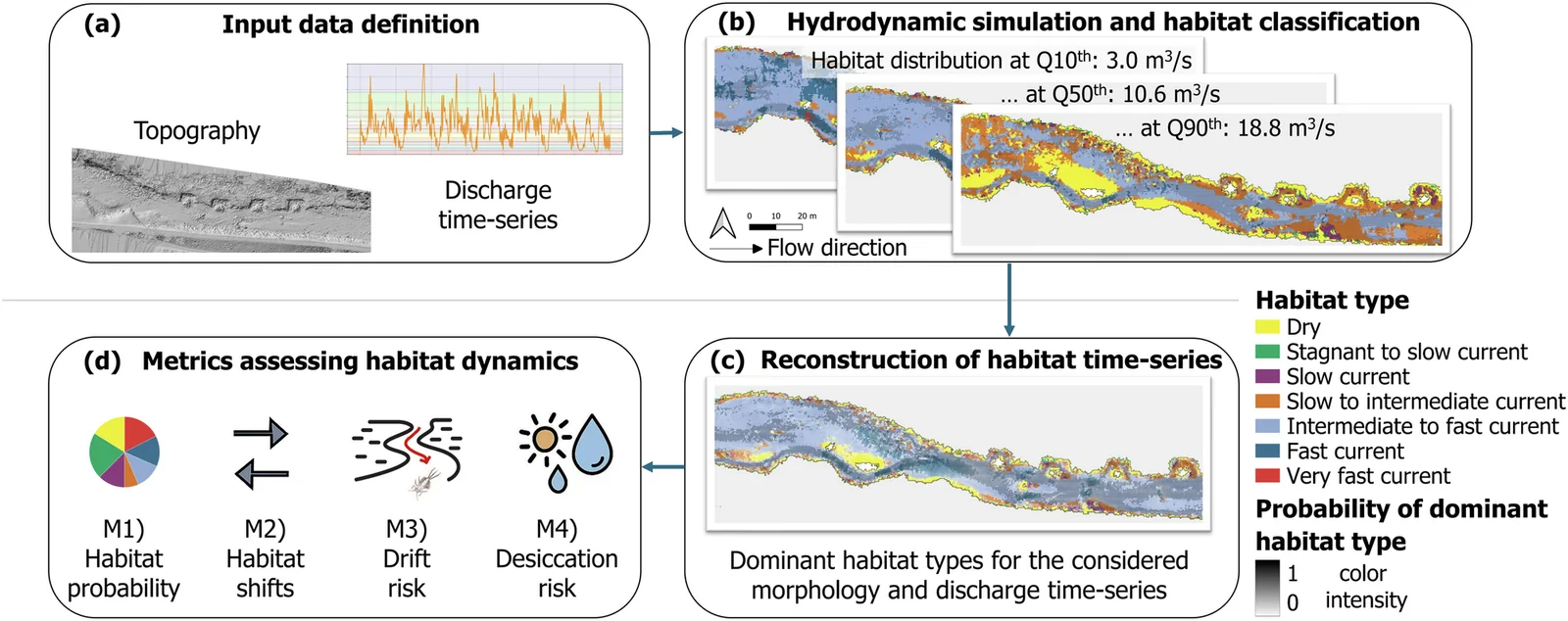
Workflow for quantifying macroinvertebrate habitat dynamics. The workflow consists of: **a** defining input data, including the discharge time-series and topography; **b** conducting hydrodynamic simulations following model calibration and validation, and classifying habitat distributions based on expert knowledge; **c** reconstructing a spatially explicit habitat time-series by combining habitat distributions with the discharge time-series; and **d** calculating patch-scale habitat metrics. The workflow is implemented in the open-source HaDy_MZB Python toolbox: https://github.com/HaDy-toolbox/HaDy_MZB

We developed a modular Python-based workflow (Fig. 1) that extends the patch-scale habitat dynamics framework of Bätz et al. (2023, 2025) by explicitly capturing hydraulic stress mechanisms relevant to macroinvertebrates. The workflow integrates discharge time-series, morphological data, hydrodynamic simulations, and ecological habitat information to generate a spatially explicit habitat time-series, from which four patch-scale metrics are derived: (M1) habitat probability, (M2) habitat shifts, (M3) drift risk, and (M4) desiccation risk. Here, a patch corresponds to a 0.5 × 0.5 m model cell, providing a practical implementation of the ecological scale relevant to macroinvertebrate larvae (Beisel et al. 2000; Béjar et al. 2020; Bätz et al. 2023).

To facilitate reproducibility and transferability of the proposed framework to other river systems and flow regimes, the workflow was implemented as an open-source Python toolbox. The toolbox, including source code, user manual, and a complete example case study, is freely available in a public GitHub repository: https://github.com/HaDy-toolbox/HaDy_MZB.

Here, we demonstrate the application of the workflow using a hydropeaked alpine reach of the Ticino River (46.515797° N, 8.688968° E), immediately downstream of a hydropower plant outlet. This case study provides a realistic setting to evaluate the workflow and interpret the resulting patch-scale metrics, rather than an engineering-grade reconstruction of site-specific hydraulic conditions. The study reach includes several instream measures along a 1 km section. Details on flow regime, morphology, hydrodynamic modelling, habitat classification, and metric calculation are provided in the following sections.

### Habitat Simulation

#### Discharge Time-Series

**Fig. 2:**
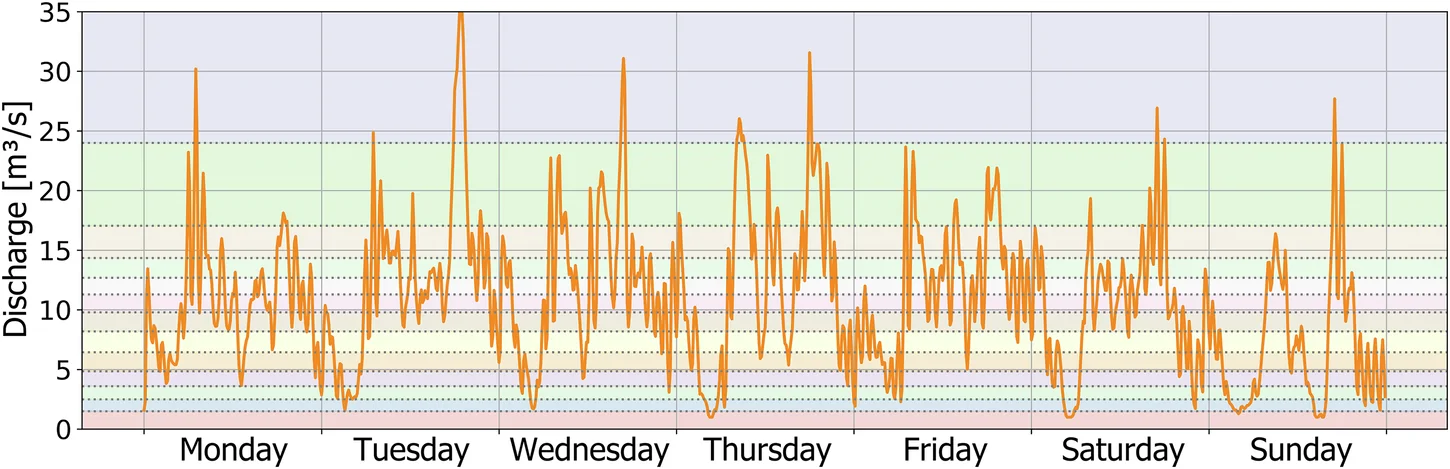
Reconstructed discharge time-series representing a typical winter hydropeaking week in an alpine river (10 min. resolution). Colored bands indicate the discharge ranges used to assign each time step to one of the 13 steady-state hydrodynamic simulations. Dashed horizontal lines indicate the boundaries between adjacent discharge ranges, with each range represented by the central simulated discharge value

The workflow requires a temporally highly resolved discharge time-series (here at 10 min resolution). For the case study, we reconstructed a representative winter hydropeaking week for an alpine river (Fig. 2). We focused on winter because hydropeaking hydrology is most pronounced during this period compared to natural discharges (i.e. mostly stable and low).

Discharge data from 20th January to 26th February 2025 were obtained from the gauging station 2494 Pollegio Campagna (FOEN 2025), located 25 km downstream of the study reach. Although this station integrates the effects of multiple hydropower plants, it exhibits a hydropeaking frequency (3–5 peaks per day) typical of alpine rivers (Hayes et al. 2025).

A representative weekly discharge time-series was derived from the multi-week observation period by averaging discharge values across corresponding time steps of each week within the observation period. The resulting discharge time-series was then scaled to match the discharge characteristics of the hydropower plant. Baseflows were aligned with the 10th percentile (3 m³/s) and peak discharges with the 98th percentile (25 m³/s). Minimum discharge was further constrained using data from gauging station 2364 Piotta (FOEN 2025), located immediately upstream of the hydropower plant outlet, ensuring consistency with minimum discharge requirements for the reach.

#### River Morphology And Structural Complexity

Given the patch-scale focus of this study, a high-resolution representation of river morphology was required. The topography of the 1-km study reach was surveyed using UAV-based photogrammetry at a discharge of 10 m³/s. A DJI Mini 4 Pro was flown at an average altitude of 35 m with >80% image overlap under light cloud cover. Georeferencing accuracy was enhanced using 32 ground control points surveyed by DGNSS (horizontal/vertical accuracy: 0.02/0.02 m).

**Fig. 3:**
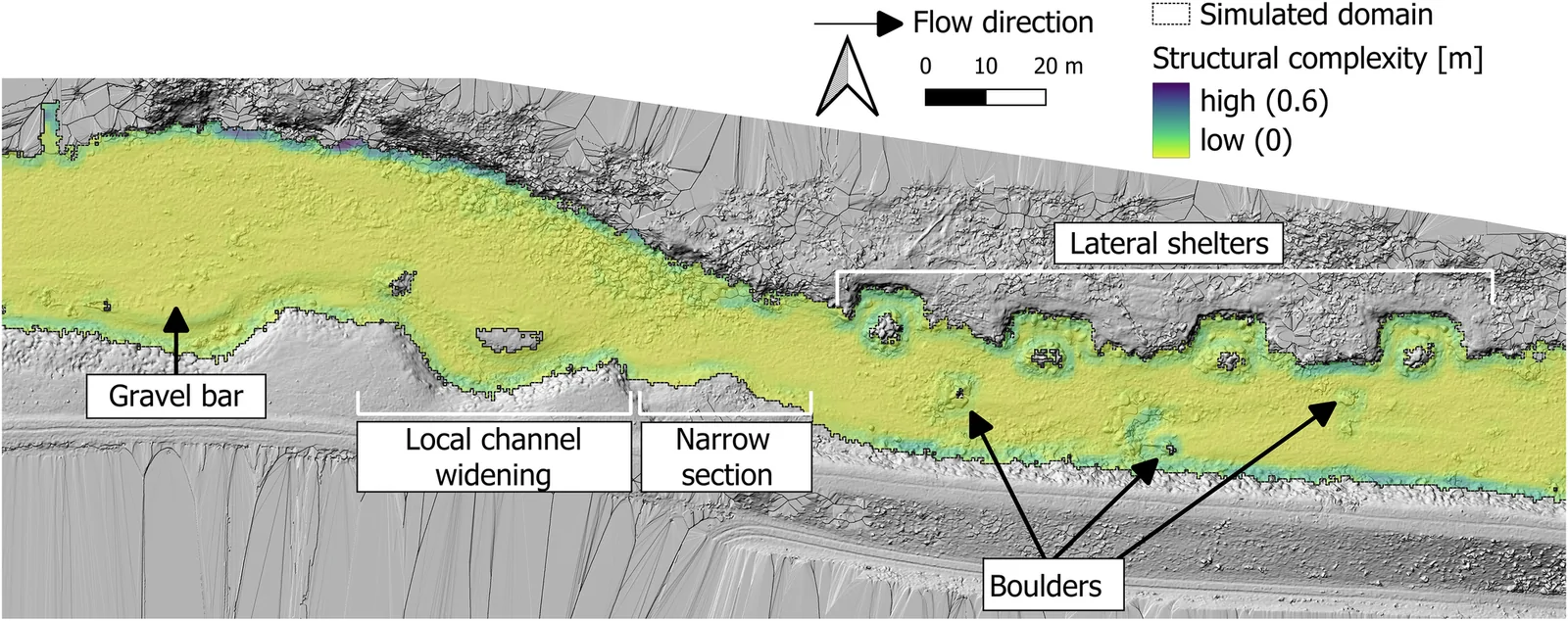
Hillshade visualization of the digital terrain model (DTM) of the Ticino study reach, with structural complexity overlaid for the simulated domain. Structural complexity is quantified as the standard deviation of bed elevation within a 5 × 5-cell moving window on the 0.5 × 0.5 m raster grid. Annotations indicate examples of morphological features and instream measures

Imagery was processed using Structure-from-Motion Multi-View Stereo reconstruction in Pix4D Mapper v4.6.4 (Pix4D SA, Switzerland), producing a Digital Terrain Model (DTM) with a pixel size of 0.013 m (Fig. 3). This DTM served as the basis for hydrodynamic simulations. Because no refraction correction was applied during photogrammetric reconstruction, submerged bed elevations may contain local depth-related uncertainty, potentially affecting absolute hydraulic accuracy. However, as the study focuses on relative spatial patterns, metric interactions, and framework performance rather than engineering-grade site-specific predictions, this limitation is expected to have limited influence on the main interpretation of habitat dynamics.

Structural complexity (Fig. 3) was quantified from the DTM after interpolation to a uniform 0.5 × 0.5 m raster grid, corresponding to the spatial resolution later used for hydrodynamic outputs and patch-scale habitat analyses. Structural complexity was calculated in QGIS as the standard deviation (STD) of bed elevation within a 5 × 5-cell moving window. This metric represents local topographic heterogeneity of the river bed, and values were interpreted comparatively rather than absolutely (Wohl 2016). The chosen window size, corresponding to an area of 2.5 × 2.5 m (6.25 m²), reflects the spatial scale at which benthic macroinvertebrate communities respond to habitat heterogeneity (Beisel et al. 2000) and aligns with the patch-scale definition used throughout the study.

The reach has an average slope of 0.01 m/m and a channel width ranging from 13 to 29 m. Although largely channelized, several local morphological features and instream measures, such as lateral shelters, boulders, and local channel widening, introduce patch-scale variability and increase structural complexity.

#### Hydrodynamic Simulations

Two-dimensional clear-water hydrodynamic simulations were conducted using BASEMENT v4.0 following standard procedures for input preparation, calibration, and validation. An unstructured triangular mesh (maximum element size ≤0.5 m) was generated from the DTM to resolve hydraulically and morphologically complex sections of the reach.

Model calibration and validation were based on 415 field measurements of water depth and current velocity at 40% of the water depth (v_40_), collected under seven discharge conditions ranging between 7.6 m^3^/s and 12.1 m^3^/s. V_40_ was used as a proxy for depth-averaged velocity, consistent with model outputs. The dataset was randomly split into equal calibration and validation subsets, and calibration was performed by iteratively adjusting a uniform Strickler roughness coefficient.

Model performance was evaluated using the Kling–Gupta Efficiency (KGE; Gupta et al. 2009), which jointly captures correlation, bias, and variability. For water depth, the model achieved a KGE of 0.79, indicating very good performance (correlation = 0.79, bias = 0.96, variability = 1.02). For current velocity, KGE was 0.47, reflecting acceptable performance, with higher agreement in bias (0.88) and variability (0.83) than in correlation (0.52).

Following calibration and validation, steady-state simulations were conducted for 13 discharge values (1.0, 2.0, 3.0, 4.2, 5.5, 7.4, 9.0, 10.6, 12.0, 13.4, 15.3, 18.8, and 29.2 m³/s), spanning the 1^st^ to 99^th^ percentile range of the reconstructed hydropeaking time-series (Fig. 2). Each discharge was simulated until hydraulic steady state was reached. Simulated water depth and velocity fields from the unstructured mesh were then interpolated onto the same uniform 0.5 × 0.5 m raster grid used for the structural-complexity analysis, ensuring a consistent spatial framework for subsequent patch-scale habitat analyses.

#### Habitat Definition and Classification

Habitat types relevant for macroinvertebrates (Table 1) were defined following the expert-based classification proposed by Tonolla (2023). This generalized, community-level classification synthesizes hydraulic preferences across a wide spectrum of macroinvertebrate taxa, life stages, and functional groups, thereby reducing the uncertainties and extensive parameterization associated with taxon-specific preference curves. The classification is grounded in empirical evidence on macroinvertebrate distributions along current-velocity gradients and has been validated through expert judgment and field observations, providing a robust and transferable basis for patch-scale habitat assessment.

Two additional types were added to include habitats considered unsuitable for most macroinvertebrates: a “dry” habitat type for patches with water depth <1 cm and an “inappropriate” habitat type for patches with very fast current velocities ( > 2.5 m/s), where colonization is physically constrained.

**Table 1:** Macroinvertebrate habitat classification at community-level (adapted from Tonolla 2023)

Category	Color	Current velocity	Habitat type
Dry		Less than 1 cm of water	-
Limnophile (lip)		Stagnant to slow current v₄₀ < 0.05 m/s	1
Limno-rheophile (lrp)		Slow current 0.05 ≤ v₄₀ < 0.25 m/s	3
Rheo-limnophile (rlp) and rheophile (rhp)		Slow to intermediate current 0.25 ≤ v₄₀ < 0.75 m/s	5
Rheobionte (rhb)		Intermediate to fast current 0.75 ≤ v₄₀ < 1.5 m/s	4
Rheobionte+ (rhb + )		Fast current 1.5 ≤ v₄₀ < 2.5 m/s	2
Inappropriate (inap)		Very fast current v₄₀ ≥ 2.5 m/s	-

Habitat types are ranked according to expected suitability for macroinvertebrates, ranging from 1 (lowest expected suitability) to 5 (highest expected suitability). Colors correspond to the habitat types shown in figures.

**Table 2:** Overview of the four patch-scale metrics used to quantify macroinvertebrate habitat dynamics

Metric	Definition	Calculation	Range	Relevance
M1) Habitat probability	Likelihood of different habitat conditions within a patch through time (Bätz et al. 2025).	${\rm{M}}1=\frac{\sum {{\boldsymbol{t}}}_{{\boldsymbol{H}}}}{{\boldsymbol{T}}}$		- Time-integrated habitat availability; identifies dominant habitat types
M2) Habitat shifts	Daily number of times (i.e., frequency) that habitat conditions change to another suitable habitat type in a patch (Bätz et al. 2025).	${\rm{M}}2=\frac{\sum {\rm{S}}}{T}$		- Indicates habitat stability and temporal persistence
M3) Drift risk	Risk of macroinvertebrate drift based on current velocity and up-ramping rate experienced within each patch over time.	M3 = P^90^ (R_drift_)		- Identifies exposure to critical passive drift conditions
M4) Desiccation risk	Risk of egg desiccation linked to hydropeaking-induced water level variations.	M4 = max (R_des_)		- Identifies exposure to critical desiccation duration

For each metric, the table summarizes its conceptual definition, calculation logic, value range, and ecological relevance

*t*_H_ duration of occurrence of a specific habitat type over the time-series, *T* total duration of the time-series, *S* habitat-shift between two consecutive time steps, *P*^90^ 90^th^ percentile value over the time-series, *max* maximum value over the time-series, *R*_drift_ drift risk as defined in Table 3, *R*_des_ desiccation risk as defined in Table 4

**Table 3:** Macroinvertebrate drift risk thresholds and corresponding metric values for the two selected habitat types

Metric value (R_drift_)	Drift risk class	Habitat type		
		Slow^a^		Slow to intermediate^b^
		Current velocity threshold	Up-ramping rate	Current velocity threshold
1	Low	v_40_ ≤ 0.4 m/s	Not relevant	v_40_ ≤ 1.2 m/s
2	Moderate	0.4 < v_40_ < 0.7 m/s	Not relevant	1.2 < v_40_ < 2.1 m/s
		0.7 ≤ v_40_ ≤ 1 m/s	< 1 cm/min	
3	High	0.7 ≤ v_40_ ≤ 1 m/s	≥ 1 cm/min	2.1 ≤ v_40_ ≤ 3 m/s
4	Very high	v_40_ > 1 m/s	Not relevant	v_40_ > 3 m/s

^a^thresholds taken from the flume experiments in Tonolla et al. (2019) and incorporating findings from Friese et al. (2025).

^b^thresholds mathematically extrapolated from the “slow” current habitat, by proportionally adjusting the upper limit of the typical current velocity range. Up-ramping rate was not included to avoid introducing additional assumptions.

**Table 4:** Macroinvertebrate desiccation risk thresholds and corresponding metric values based on Kennedy et al. (2016) and Jaulin Gautronneau et al. (2026)

Metric value (R_des_)	Desiccation risk class	Max. dry duration
1	Low	≤ 1 h
2	Moderate	1–5 h
3	High	5–9 h
4	Very high	> 9 h

The classification was applied to hydrodynamic simulations of the 13 steady-state discharges, assigning each 0.5 × 0.5 m raster cell to a habitat type based on simulated water depth and current velocity. Habitat availability across discharges and example habitat maps for the 10^th^, 50^th^, and 90^th^ discharge percentiles are provided in Supplementary information 1.

#### Habitat Time-Series

To study habitat dynamics at the patch scale, habitat classifications from the steady-state hydraulic simulations (i.e. spatial distributions) were combined with the recorded discharge time-series (i.e. temporal forcing) to reconstruct a time-explicit sequence of habitat conditions. Their integration yields a habitat time-series that reflects the patch-scale habitat dynamics experienced by macroinvertebrates during a typical winter hydropeaking week in an alpine environment.

To do so, the discharge time-series was divided into discharge ranges (marked with different colors in Fig. 2) each bounded by the midpoint between two adjacent simulated discharge values. Each discharge range was assigned the habitat distribution of its midpoint value (e.g. habitat distribution of 12.0 m³/s representing discharges between 11.3 and 12.7 m³/s), assuming comparable hydraulic conditions within each interval. This approach preserves temporal variability in the hydropeaking signal while maintaining ecological and hydraulic consistency at the patch scale.

The resulting habitat time-series provides the basis for calculating all patch-scale metrics.

### Metrics Description and Computation

The following macroinvertebrate patch-scale habitat metrics (hereafter referred to as metrics) were derived from the habitat time-series to quantify complementary ecological facets of habitat dynamics experienced by macroinvertebrates (Table 2). Habitat probability (M1) and shifts (M2) describe the availability and temporal persistence of suitable conditions and are therefore generally related to habitat conditions, whereas drift (M3) and desiccation (M4) metrics represent exposure to harmful hydraulic events with associated ecological risk.

The analysis below focuses on habitat types characterized by “slow” and “slow to intermediate” current (Table 1), hereafter referred to as “slow” and “slow to intermediate” habitats. These habitats represent lentic to weakly lotic conditions preferred by many benthic macroinvertebrates and are highly susceptible to passive drift (Schülting et al. 2023; Friese et al. 2025) and to desiccation or stranding (Tonolla et al. 2023; Jaulin Gautronneau et al. 2026). Because drift and desiccation thresholds are habitat-specific and well defined for these habitat types, they provide a robust ecological basis for developing and interpreting the patch-scale metrics.

#### Metric 1: Habitat Probability Within Patches

The first metric represents the patch-specific probability of experiencing different habitat types over the entire time-series. It reflects the cumulative duration of a habitat within a patch, thereby quantifying spatio-temporal habitat availability (Bätz et al. 2025). Ecologically, this metric captures the set of habitat conditions (i.e availability and dominance) macroinvertebrates face under hydropeaking regimes.

To compute this metric, the duration of occurrence of each habitat in the habitat time-series is summed for each patch and then divided by the total length of the time-series. This results in a probability between 0 (lowest likelihood) and 1 (highest likelihood) for each habitat type, with the patch-wise probabilities across all habitat types summing to 1.

#### Metric 2: Habitat Shifts Within Patches

The second metric quantifies the average number of daily shifts between different suitable habitat types within each patch. It reflects patch-scale habitat persistence and temporal stability, which are key ecological aspects for macroinvertebrates due to their limited mobility with respect to the rapid hydraulic changes induced by hydropeaking (Bätz et al. 2025).

To compute this metric, every time the habitat changes from one suitable habitat type to another is counted for each patch across the entire habitat time-series. The total number of these shifts is then divided by the number of simulation days. The resulting value ranges from 0, when the habitat remains stable throughout, to the theoretical maximum defined by the number of time steps. Higher values indicate low temporal persistence and more dynamic habitat conditions.

#### Metric 3: Drift Risk

The third metric addresses the risk of passive macroinvertebrate drift for the “slow” and “slow to intermediate” habitats (Table 3). Drift-risk thresholds do not represent species-specific tolerance limits, but rather community-level approximations reflecting the average hydraulic sensitivity of macroinvertebrate assemblages associated with different habitat types defined by Tonolla (2023).

For the “slow” habitat, risk classes are defined using thresholds for current velocity and up-ramping rate, based on empirical studies (Tonolla 2023; Schülting et al. 2023). For the “slow to intermediate” habitat, equivalent empirical thresholds are currently lacking. Current-velocity thresholds were therefore extrapolated by preserving the proportional relationship between the empirical drift-risk thresholds defined for the “slow” habitat and the upper velocity limit of this habitat type (Tonolla 2023) and then applying the same relationships to the upper velocity limit of the “slow to intermediate” habitat. Up-ramping rate was omitted to avoid introducing additional unsupported assumptions.

Drift risk is computed for every 10-minute time step, reflecting experimental findings that drift peaks within minutes directly after abrupt increases in current velocity (Bruno et al. 2010). For each patch, the 90^th^ percentile of all drift-risk values is retained as the final metric drift risk class. This choice reflects frequent exposure to elevated drift conditions while minimizing the influence of isolated extreme events.

#### Metric 4: Desiccation Risk

Desiccation risk is addressed as the maximum consecutive duration each patch remains dry over the entire habitat time-series, under the assumption that longer dry periods result in lower macroinvertebrate egg viability. Similar to the drift-risk metric, desiccation thresholds do not represent species-specific tolerance limits but provide a generalized community-level approximation of hydropeaking-related desiccation stress. The selected thresholds (Table 4) are based on empirical findings from Kennedy et al. (2016) and Jaulin Gautronneau et al. (2026). Both studies highlight that even single desiccation events can already drastically reduce egg survival, representing a critical bottleneck for macroinvertebrate persistence.

For each patch, consecutive dry periods are aggregated to identify uninterrupted drying events. The final metric corresponds to the maximum consecutive dry duration experienced by each patch (i.e. the most critical event), providing an ecologically meaningful indicator of macroinvertebrate egg exposure to desiccation stress. Because the metric is based on maximum consecutive dry duration, increased hydropeaking frequency may shorten uninterrupted drying periods and thereby reduce desiccation risk in some patches.

#### Metric Relationships and Interactions

In the final analytical stage, interactions among the patch-scale metrics were examined using two complementary approaches to study both pairwise relationships and combined behavior under hydropeaking conditions. First, pairwise relationships between metrics were quantified using Spearman rank correlation coefficients, which capture the strength and direction of monotonic relationships without assuming linearity. Scatterplots were used to visually explore these relationships and identify potential non-linear patterns.

Second, to synthesize the combined information contained in the four metrics, patches belonging to the same habitat type were clustered using the KMeans implementation in the sklearn.cluster Python package (Pedregosa et al. 2011). The purpose of clustering was to group patches with similar multimetric profiles, thereby summarizing complex combinations of macroinvertebrate habitat conditions and risk into a reduced number of ecologically interpretable groups. Because each metric represents a distinct dimension of macroinvertebrate habitat dynamics, clustering enables the identification of patterns that are not apparent when examining each metric separately. The four patch-scale metrics were used as clustering variables. Because these variables differed substantially in scale and range, all metrics were standardized to zero mean and unit variance (z-score normalization) prior to clustering to ensure equal weighting in the Euclidean distance calculations underlying the K-means algorithm.

The optimal number of clusters was determined using the silhouette score, following standard practice for evaluating cluster separation and interpretability (Rousseeuw, 1987). Cluster analysis was conducted separately for each of the two selected habitat types to account for differences in hydraulic tolerance and ecological relevance. Cluster solutions ranging from two to nine clusters were evaluated, and the solution yielding the highest silhouette score was retained for subsequent analyses (see Supplementary information 2). A fixed random seed (random_state = 0) was used to ensure reproducibility of the clustering results.

As for the differences in the distribution of habitat probabilities among habitat types, differences in the distribution of structural complexity among clusters were studied using the non-parametric Kruskal–Wallis test. When significant differences were detected (*p* < 0.05), pairwise comparisons were performed using Dunn’s post hoc test with Holm correction for multiple testing.

Linking the clustering to structural complexity enables the investigation of how multimetric macroinvertebrate habitat conditions relate to river morphology.

## Results on Metrics Performance

### Simulated Habitats

**Fig. 4:**
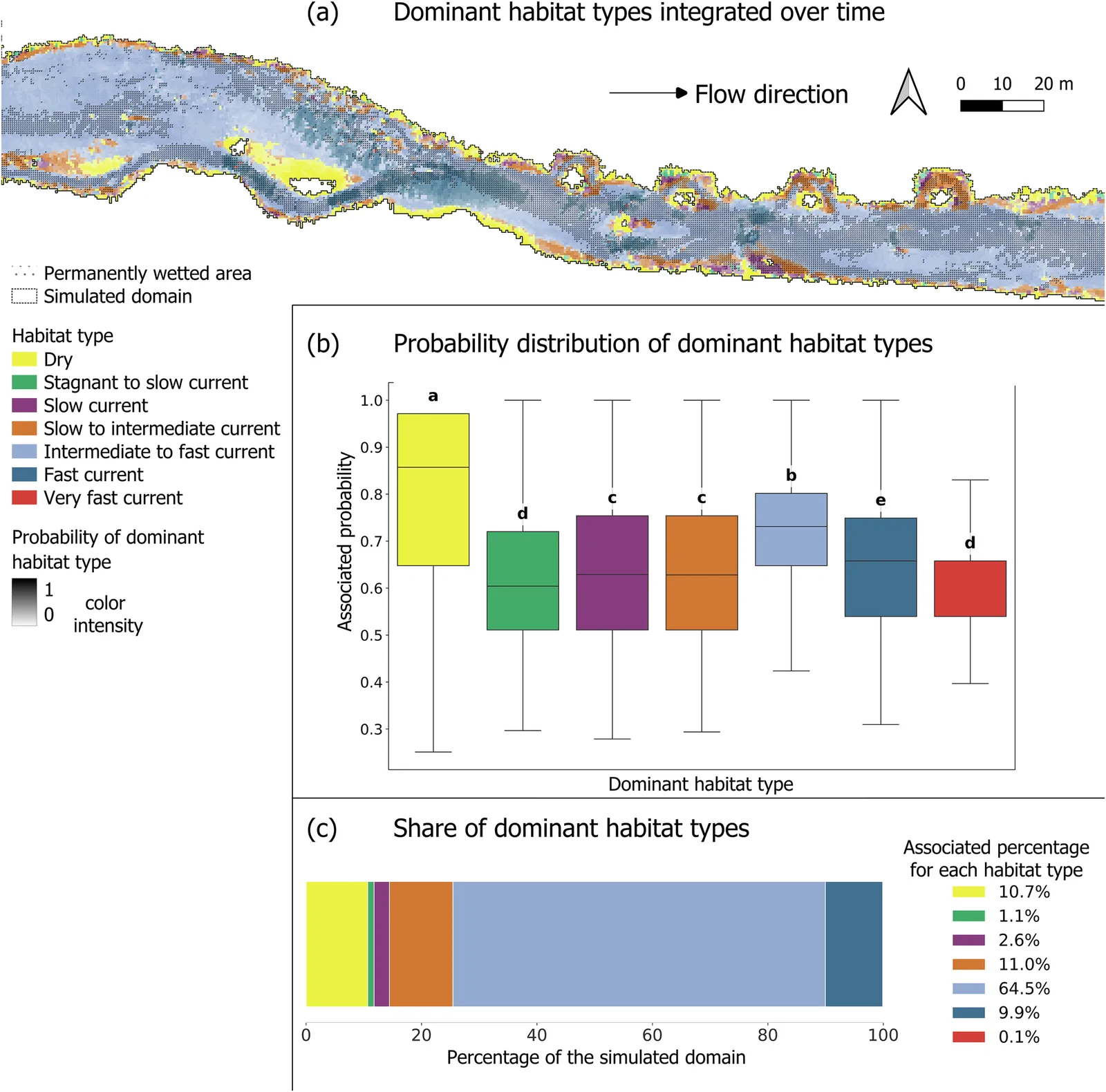
Dominant macroinvertebrate habitat types over the full habitat time-series. Colors indicate habitat types consistently across all panels. **a** Spatial distribution of dominant habitat types integrated over the time-series, with color intensity representing the patch-specific probability of occurrence. **b** Probability distributions associated with the dominant habitat types across all patches. Boxes show the median (line) and interquartile range; whiskers indicate minimum and maximum non-outlier values; circles denote outliers. Differences among habitat types were tested using a Kruskal–Wallis test (*H* = 5822; *p* < 0.05), followed by Dunn–Holm post hoc comparisons. Letters above the boxplots indicate statistically homogeneous groups; habitat types sharing a letter do not differ significantly. **c** Proportional share of each dominant habitat type across the simulated domain, with percentage values annotated

To provide an overview of the spatio-temporal integrated habitat availability during a typical winter hydropeaking week in an alpine river, Fig. 4 summarizes the dominant habitat types for macroinvertebrates. Here, the dominant habitat type for each patch is defined as the habitat type with the highest probability over the habitat time-series.

The spatial pattern (Fig. 4a*)* shows that patches dominated by “stagnant”, “slow”, or “slow to intermediate” current habitats occur primarily along the riverbanks and in patches influenced by local instream measures. These areas also exhibit strong patchiness, driven by rapid spatial alternation in dominance among the three habitat types and dry conditions. In contrast, the main channel is more homogeneous and is consistently characterized by the dominance of “intermediate to fast” to “fast” habitats.

The probability associated with dominant habitat types (Fig. 4b) shows that dry conditions have the highest overall probability and differ significantly from the other habitat types. This is because many patches located at higher elevations are only inundated during the highest discharges (see Supplementary information 1). As a result, higher elevated patches of the modeled domain (i.e. wetted area at the 99^th^ percentile discharge) remain dry for most of the week (see also Fig. 2). Among the suitable habitat types, “intermediate to fast” exhibit a significantly higher probability compared to the other habitat types, consistent with its spatial predominance within the main channel.

The proportional spatial distribution of each dominant habitat type across the simulated domain (Fig. 4c) confirms these observations, with “stagnant”, “slow”, and “slow to intermediate” habitats collectively accounting for only 14.7% of the simulated domain, whereas the “intermediate to fast” habitat is dominant in 64.5% of all patches.

### Metric Results

The following subsections present the outcomes for the four metrics for all patches that experience “slow” or “slow to intermediate” habitat types at least once in the habitat time-series. Analyses focus on these habitat types because they represent ecologically relevant habitats for benthic macroinvertebrates that are particularly susceptible to hydropeaking-related drift and desiccation, and for which habitat-specific ecological thresholds provide a robust basis for interpreting the four proposed metrics.

**Fig. 5:**
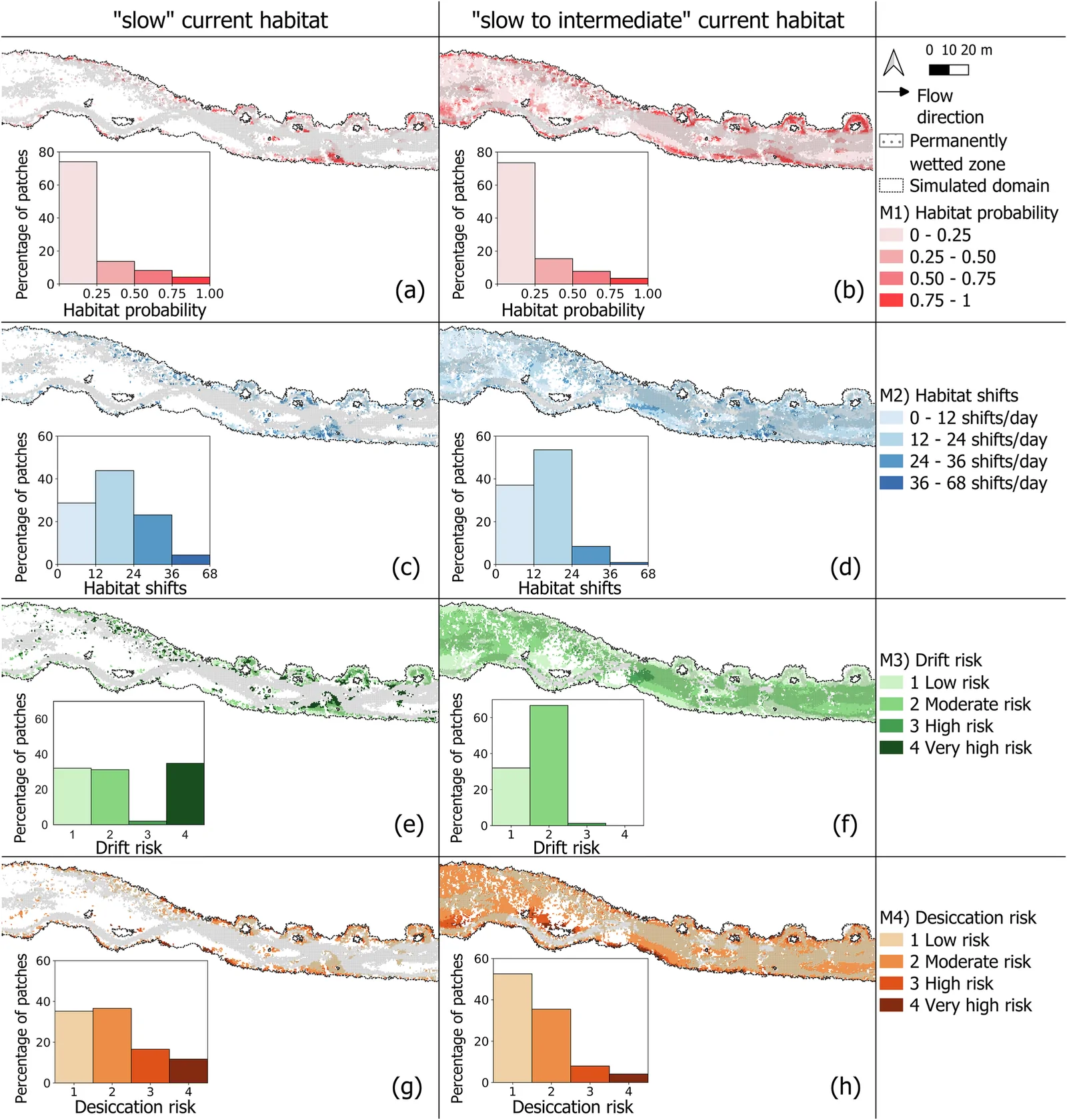
Spatial distribution of macroinvertebrate patch-scale metrics for a selected section of the study reach, showing **a**, **b** habitat probability, **c**, **d** habitat shifts, **e**, **f** drift risk, and **g**, **h** desiccation risk. The columns show results for patches that experience the “slow” current habitat (left) or “slow to intermediate” current habitat (right) at least once in the habitat time-series. Each map is accompanied by a histogram showing the distribution of metric values as percentages of all patches experiencing the respective habitat at least once during the habitat time-series

An overview of the corresponding metric results is provided in Fig. 5. Across the simulated domain, approximately 17% of patches experience the “slow” habitat, whereas about 81% of patches experience the “slow to intermediate” habitat at least once during the habitat time-series.

#### M1: Habitat Probability

Although the probability distributions for the two selected habitat types appear broadly similar in the histograms (Fig. 5a and b), their spatial patterns differ considerably. Patches experiencing the “slow” habitat are comparatively few and are mainly located along riverbanks, on bars, and around instream measures such as lateral shelters and large boulders. In contrast, the “slow to intermediate” habitat is more widely distributed, with higher probabilities along the riverbanks and markedly lower values within the main channel.

Across the modelled domain, most patches show probabilities below 0.25 for both habitat types, with 74% of patches in the “slow” habitat and 73% in the “slow to intermediate” habitat falling within this range. This indicates that lentic to weakly lotic macroinvertebrate habitats are not widely available under typical winter hydropeaking conditions. This low persistence stems from rapid and frequent discharge fluctuations, which substantially increases habitat dynamics.

#### M2: Habitat Shifts

Among the patches experiencing either of the two considered habitat types, most undergo 12–24 habitat shifts per day (Fig. 5c and d). This corresponds to a change in suitable habitat conditions roughly every 30–60 min, highlighting the high temporal variability to which these patches, and the macroinvertebrates living in them, are exposed under hydropeaking.

For patches associated with the “slow” habitat, the next most represented categories are 0–12 and 24–36 shifts per day, which occur roughly in equal proportions. In contrast, for the “slow to intermediate” habitat, 0–12 shifts per day is the clear second most frequent category, while 24–36 shifts per day is comparatively rare.

Spatially, higher numbers of habitat shifts occur for both habitat types near instream measures such as large boulders in the main channel, along the margins of lateral shelters, and along river bars. Patches experiencing the “slow to intermediate” habitat within the main channel generally show lower shifts.

Habitat shifts towards dry or exceeding specific current-velocity thresholds are examined in detail in the following two sections through the drift and desiccation risk metrics.

#### M3: Drift Risk

The two habitat types exhibit distinctly different drift-risk distributions (Fig. 5e and f). For patches associated with the “slow” habitat, drift risk shows a trimodal pattern, with most patches falling into risk class 1 (low), class 2 (moderate) or risk class 4 (very high). Only few patches fall into risk class 3 (high) for this habitat. In contrast, patches experiencing the “slow to intermediate” habitat are predominantly assigned to risk class 2 (moderate), followed by risk class 1 (low). The highest drift-risk value (class 4) does not occur for this habitat, reflecting its greater tolerance to elevated current velocities.

Spatially, both habitat types exhibit a consistent trend of increased drift risk towards the main channel, particularly in sections where the channel narrows and hydraulic forcing intensifies. This gradient is notably stronger for the “slow” habitat, where patches adjacent to the main channel can reach very high drift-risk values (class 4).

#### M4: Desiccation Risk

The two selected habitats show partly similar desiccation-risk distributions (Fig. 5g and h), although differences emerge at higher risk levels. For patches associated with the “slow” current habitat, a noticeably larger proportion falls into risk classes 3 (high) and 4 (very high), with fewer patches in risk class 1 (low). In contrast, patches experiencing the “slow to intermediate” habitat tend to fall within the low to moderate desiccation risk, with markedly few reaching the highest risk classes.

Desiccation risk is elevated along riverbanks, around emergent boulders associated with instream measures, and across gently sloped bar surfaces—patches particularly prone to drying during low-discharge periods. Narrower channel sections are generally less affected, reflecting their more persistent wetting.

### Interactions between Metrics

#### Pairwise Interactions Between Metrics

**Fig. 6:**
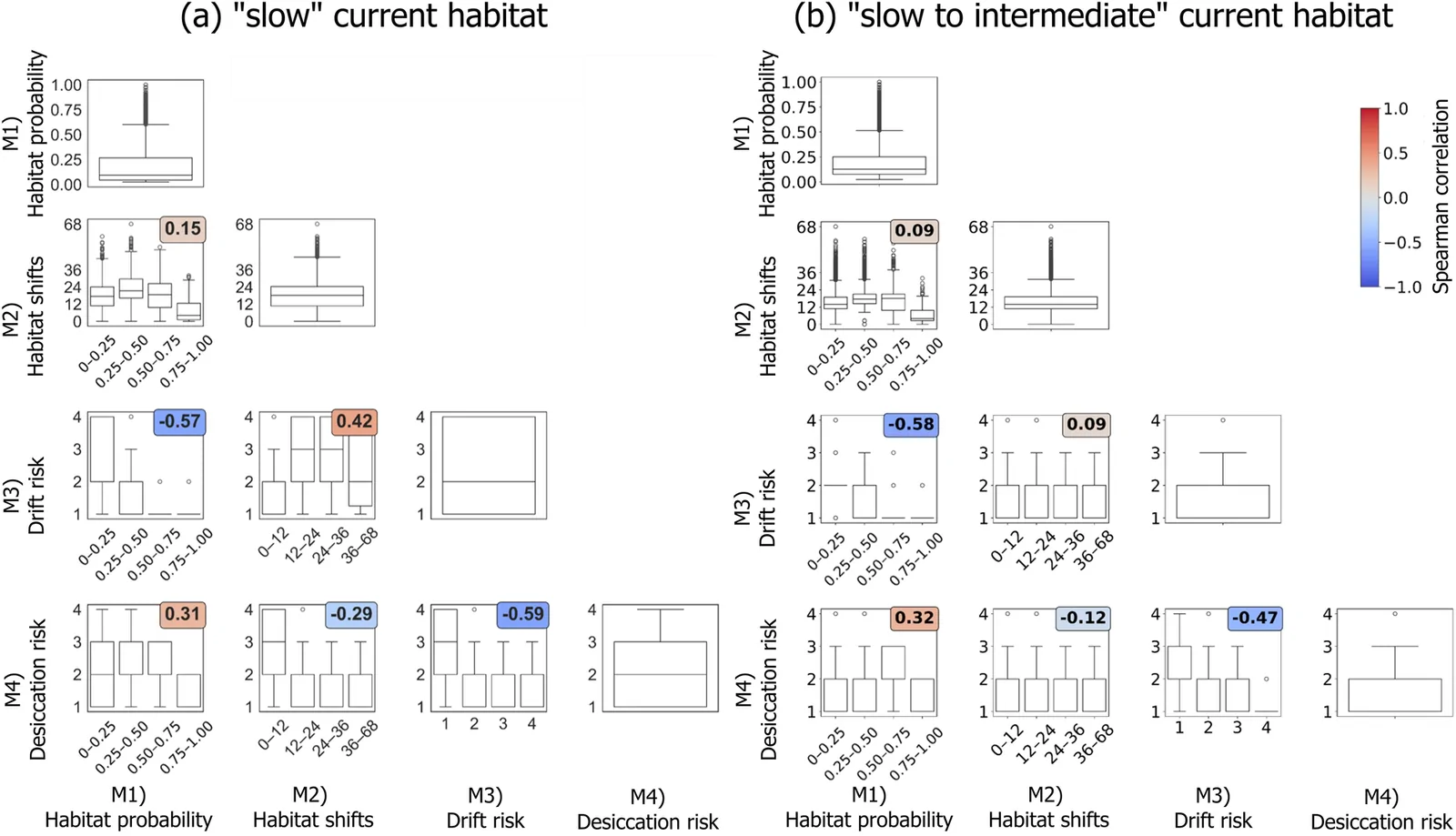
Pairwise relationships among the four macroinvertebrate-specific patch-scale metrics for **a** the “slow” current habitat and **b** the “slow to intermediate” current habitat. The panels show the distributions of metric values and their pairwise associations; univariate distributions are shown on the diagonal. Boxes show the median (line) and interquartile range; whiskers indicate minimum and maximum non-outlier values; circles denote outliers. When only a line appears (e.g., for drift risk), values are strongly concentrated within a single risk class

The interactions among metrics are shown in Fig. 6. These pairwise relationships provide insight into how different dimensions of habitat dynamics co-vary and where metrics capture complementary information.

A negative correlation is observed between habitat probability and drift risk for both habitat types, indicating that patches where “slow” or “slow to intermediate” habitats occur more persistently tend to experience lower drift risk. However, a substantial portion of the variance ( ≈ 40%) remains unexplained, demonstrating that some patches with low habitat persistence may still show low drift risk. This suggests that habitat probability alone cannot fully predict drift susceptibility.

Correlations involving habitat shifts are generally weak ( < 0.15), reflecting the multidimensional nature of habitat dynamics. Slightly stronger correlations appear for the “slow” habitat between habitat shifts and drift risk (0.42) and between habitat shifts and desiccation risk (–0.29), though these values remain low. Interpretation of intermediate drift-risk categories for the “slow” habitat is limited because only a small number of patches fall into these categories, resulting in non-representative distributions (see histograms in Fig. 5).

A negative correlation also emerges between drift risk and desiccation risk for both considered habitat types, but again roughly half of the variance is unrelated. This indicates that patches exposed to high current velocities (high drift risk) are not necessarily those that dry most frequently and vice versa.

Overall, the correlations reveal consistent qualitative trends across the two selected habitat types; however, the generally weak to moderate effect sizes indicate that no single metric pair dominates the explanation of patch-scale habitat dynamics. This remaining unexplained variance highlights the need to explore how combinations of all four metrics jointly characterize patch-scale habitat dynamics using a cluster analysis.

#### Multivariate Interactions Among Metrics

**Fig. 7:**
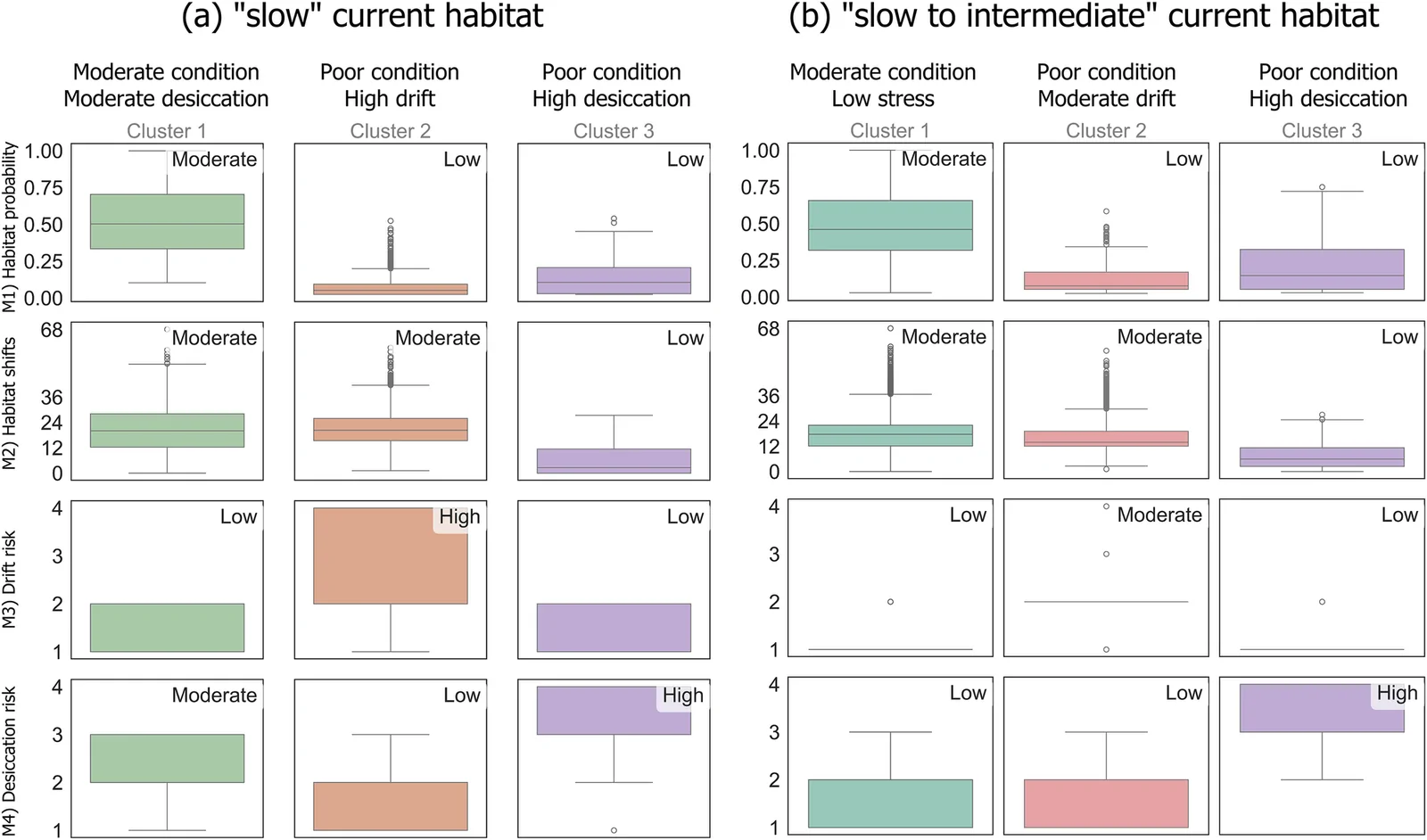
Distribution of macroinvertebrate patch-scale metric values across the three clusters identified for **a** the “slow” current habitat and **b** the “slow to intermediate” current habitat. Boxplots show the median (line), interquartile range (boxes), and minimum and maximum values excluding outliers (whiskers); circles indicate outliers. When only a line is visible, values are highly concentrated within a single risk class. Cluster labels provide interpretative summaries of overall habitat condition (habitat probability and habitat shifts) and prevailing risk (drift risk or desiccation risk). Colors are consistent for clusters with similar metric profiles

Based on silhouette scores, three clusters were identified as the optimal solution for both habitat types (Supplementary information 2), yielding mean silhouette scores of 0.40 for the “slow” habitat and 0.49 for the “slow to intermediate” habitat. Figure 7 summarizes the distribution of metric values within each cluster, which were subsequently interpreted and labelled according to their overall habitat condition (i.e. habitat type probability and shifts) and prevailing risk (i.e. drift and desiccation).

For both considered habitat types, Cluster 1 (“Moderate condition”) represents patches with moderate habitat probability and intermediate habitat shifts, corresponding to the most stable habitat conditions for macroinvertebrates under hydropeaking. Associated risk levels remain generally low to moderate, with desiccation representing the dominant stressor in the “slow” habitat, while overall levels of stress remain low in the “slow to intermediate” habitat.

In contrast, Clusters 2 and 3 (“Poor condition”) are primarily characterized by very low habitat probabilities. However, the clusters differ in their dominant stressor. Cluster 2 is associated with elevated drift risk exposure, with patches showing moderate to high drift risk, while Cluster 3 is consistently characterized by high desiccation risk. Drift risk is more pronounced in Cluster 2 of the “slow” habitat than in the “slow to intermediate” habitat.

Despite similarities in general cluster structure between the two habitat types, the relative prominence of drift versus desiccation risk differs, reflecting their distinct spatial occurrence and hydrodynamic exposure.

**Fig. 8:**
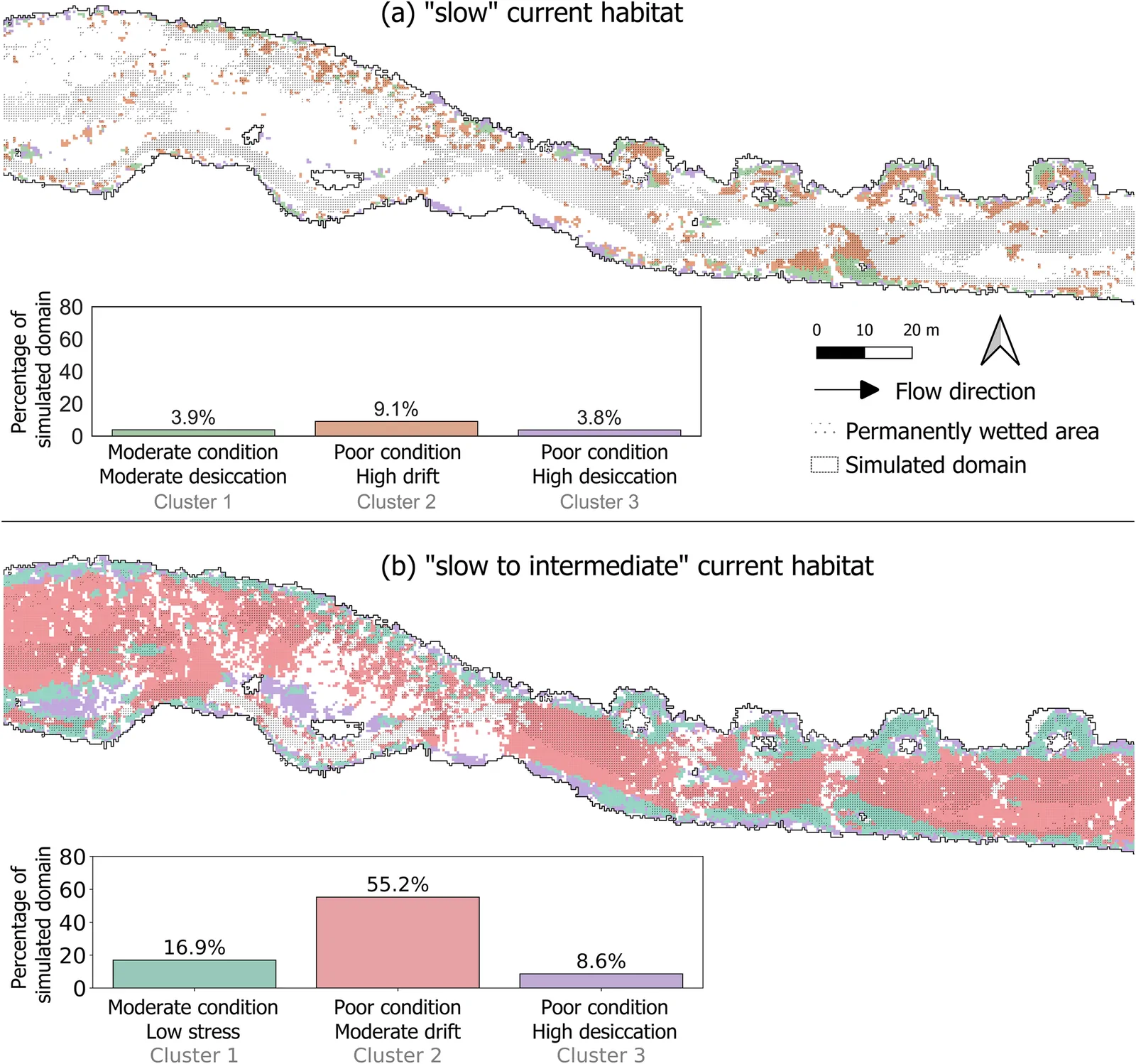
Spatial distribution of clusters for the two selected macroinvertebrate habitat types: **a** “slow” current habitat and **b** “slow to intermediate” current habitat. Colors correspond to clusters defined in Fig. 7. The accompanying bar plots show the proportion of the total simulated domain represented by patches assigned to each cluster

From a spatial perspective (Fig. 8), both habitat types exhibit broadly consistent patterns in how clusters are arranged across the reach. Cluster 2, characterized by poor condition and moderate to high drift risk, is concentrated within or near the main channel, especially in narrower sections where hydraulic forcing intensifies quickly with increasing discharges.

In contrast, Cluster 3 (“Poor condition / High desiccation”) is primarily located along channel margins, including gently sloping banks and bars in the wider river sections. Patches in these areas may show prolonged drying during hydropeaking, leading to an increased risk of desiccation for macroinvertebrate eggs.

Cluster 1, which represents patches with moderate habitat condition and the lowest overall risk levels, occupies intermediate zones between high-current areas in the main channel and the drying-prone exposed channel margins. For the “slow” habitat (Fig. 8a), Cluster 1 is often found on shallow bars near the riverbanks and in sheltered patches behind boulders, or within shallower areas inside lateral shelters. For the “slow to intermediate” habitat (Fig. 8b), a similar spatial pattern emerges, but Cluster 1 is more widely distributed within lateral shelters, patches previously identified as exhibiting both low drift and low desiccation risk.

#### Metrics Response to Structural Complexity

**Fig. 9:**
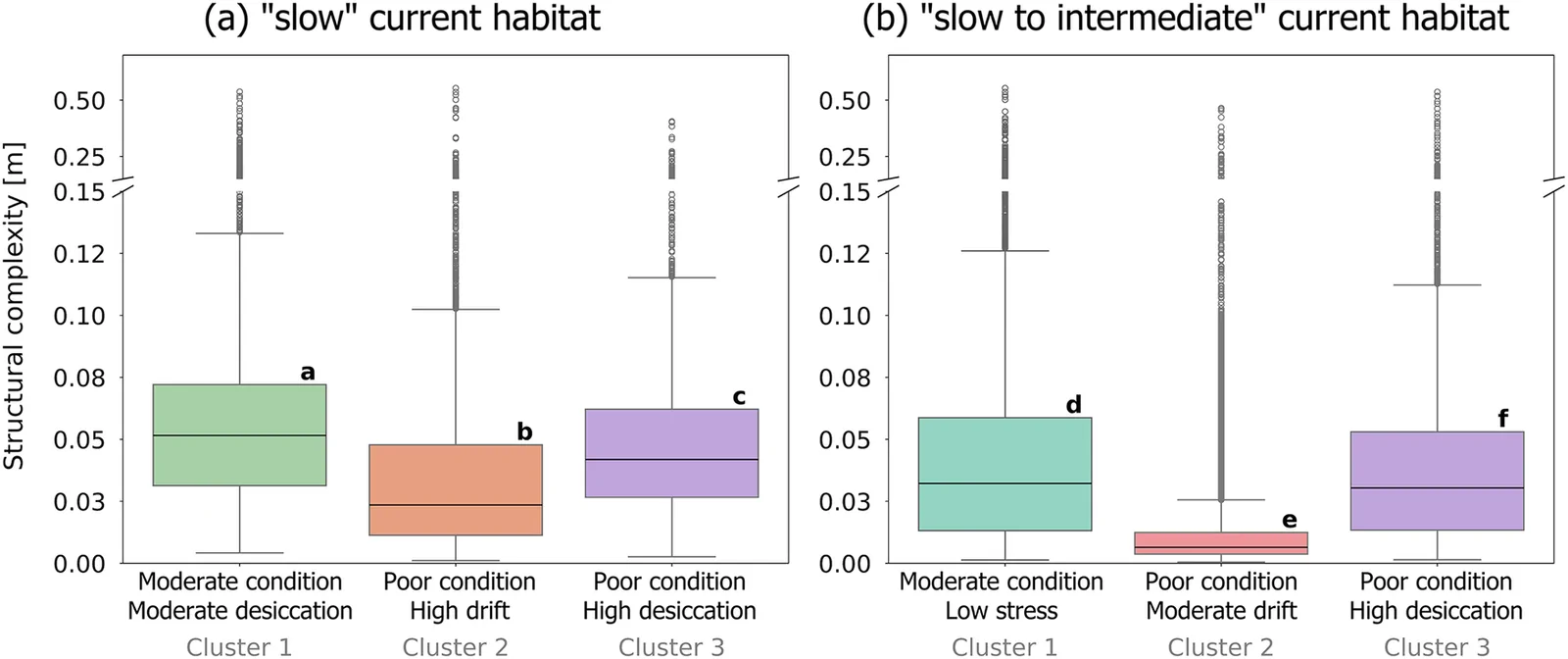
Distribution of structural complexity (i.e. the standard deviation of the surrounding bed elevation) across patch clusters for **a** the “slow” current habitat and for **b** the “slow to intermediate” current habitat. Boxplots show the median (line), interquartile range (boxes), and minimum and maximum values excluding outliers (whiskers); circles indicate outliers. Differences among clusters were tested using Kruskal–Wallis tests (*H* = 2251 for “slow” current habitat; *H* = 21 127 for “slow to intermediate” current habitat; *p* < 0.05), followed by Dunn–Holm post hoc comparisons. Letters above the boxplots indicate statistically homogeneous groups, with clusters sharing a letter not differing significantly. Note the break in the y-axis

The spatial organization of metric clusters described in the previous section shows some relationship with morphological features. Patch cluster membership was therefore related to local structural complexity, expressed as the standard deviation of bed elevation within a 2.5 m window (5 × 5-cell) surrounding each patch (Fig. 9). Results from the Dunn–Holm post hoc test indicate that structural complexity differs significantly among clusters for both habitat types, confirming that each cluster is associated with a characteristic range of morphological conditions.

For both habitat types, Cluster 1, which represents patches with the highest habitat probability and lowest overall risks, is consistently associated with locally high structural complexity, with median value of 5.2 cm for the “slow” habitat and 3.2 cm for the “slow to intermediate” habitat. It is followed by Cluster 3, defined by low habitat probability and high desiccation risk, with median structural-complexity values of 4.2 and 3.0 cm for the “slow” and “slow to intermediate” habitats, respectively. Cluster 2, characterized by low habitat probability and moderate to high drift risk, shows the lowest structural complexity, with median values of 2.4 and 0.6 cm, respectively. However, clusters associated with the “slow” habitat generally occur at higher structural complexity values than those associated with the “slow to intermediate” habitat, reflecting the stronger dependence of low-current conditions on pronounced morphological heterogeneity. An exception to this pattern is Cluster 3, which shows comparable structural complexity ranges across both habitat types and corresponds to gently sloping banks and bar surfaces along channel margins in wider river sections.

Consistent with the spatial patterns previously described, Cluster 1 occupies patches characterized by enhanced structural complexity, often created or reinforced by instream measures such as boulders and lateral shelters. In contrast, Cluster 2 is associated with the lowest levels of structural complexity. This pattern is particularly pronounced for the “slow to intermediate” habitat, where Cluster 2 occupies a narrow range of very low complexity values (interquartile range of 0.9 cm) and is largely confined to the main channel in morphologically uniform and hydraulically constrained sections of the main channel.

These results indicate that structurally complex patches tend to support combinations of habitat conditions that are associated with higher habitat persistence and lower drift/desiccation risk under hydropeaking.

## Discussion

### Performance of the Metrics

The four proposed patch-scale metrics each capture a distinct and ecologically meaningful dimension of habitat dynamics experienced by macroinvertebrates under hydropeaking.

Habitat probability (M1) represents the temporal availability of suitable hydraulic conditions, and our results highlight the scarcity of lentic to weakly lotic habitats under hydropeaking. These habitats are ecologically critical, as they typically support higher macroinvertebrate abundance and diversity, as well as specialized taxa with narrower current-velocity tolerances and a sensitive response to rapid hydraulic changes (Tonolla et al. 2023; Friese et al. 2025; Jaulin Gautronneau et al. 2026). In particular, patches associated with the “slow” habitat are generally located outside the permanently wetted area and therefore show low probabilities, primarily driven by repeated drying during low discharges rather than exposure to extreme velocities (Fig. 5a). In contrast, “slow to intermediate” habitat patches are often situated in narrower or more central channel sections and show higher probabilities, although they frequently shift toward faster habitat types during peak discharges (Fig. 5b). These patterns align with observations that channelized rivers lack sufficient morphological buffering to dampen hydraulic stress, limiting the availability of lentic to weakly lotic habitats (Vanzo et al. 2016; Bätz et al. 2025). Conversely, patches influenced by boulders, gravel bars and along river shores and secondary channels exhibit higher probabilities, indicating a somewhat greater resilience to short-term discharge variations and intermittent refuge potential (Vanzo et al. 2016; Hauer et al. 2017; Bätz et al. 2025).

Habitat shifts (M2) directly quantify temporal habitat persistence at the patch scale and complement habitat probability by describing how frequently conditions change. Similarly high shift frequencies for both “slow” and “slow to intermediate” habitats, typically one shift every 30–60 minutes, indicate that lentic to weakly lotic habitats are inherently “nervous” (sensu Bätz et al. 2023) under hydropeaking, repeatedly exposing macroinvertebrates to rapidly changing hydraulic conditions (Fig. 5c and d). Bätz et al. (2025) demonstrated that this metric responds strongly to river morphology. In narrow or channelized river reaches, shifts increase by a factor of 54 compared to natural flow regimes, whereas wider and structurally diverse morphologies show a smaller increase (factor 22). This highlights the buffering role of morphology (Shea and Peterson 2007; Vanzo et al. 2016), while also acknowledging that instream measures such as large boulders can locally increase shift frequencies (Fig. 5c and d). Such frequent shifts may explain why macroinvertebrates in hydropeaking rivers exhibit habitat preferences that differ from conspecifics in non-regulated rivers (Judes et al. 2023), as adjusting to these dynamics imposes energetic and behavioral costs, particularly for taxa with limited mobility and narrow tolerance ranges (Bätz et al. 2023). Consequently, even where partially mitigated by instream measures, high shift frequencies constitute a persistent stressor for macroinvertebrates under hydropeaking.

Drift risk (M3) captures exposure to abrupt increases in current velocity, a well-established trigger of passive macroinvertebrate drift under hydropeaking (Bruno et al. 2010, 2016; Naman et al. 2016; Tonolla et al. 2023). Consistent with previous studies (Vanzo et al. 2016; Hauer et al. 2017; Bätz et al. 2025), high drift risk is generally concentrated near and within the main channel, where discharge refugia are scarce and hydraulic forcing increases rapidly with discharge (Fig. 5e and f). In contrast, patches located on gently sloping banks or within sheltered zones associated with instream measures exhibit lower drift risk. Beyond this general pattern, our results reveal a high-risk–small-area mechanism: “slow” habitats occupy only a small fraction of the simulated domain (17% versus 81% for “slow to intermediate”), yet account for a disproportionate share of very high drift-risk values. Experimental evidence shows that macroinvertebrate communities in lentic habitats respond more strongly to hydropeaking, with drift intensity driven primarily by absolute current velocity (Friese et al. 2025). Because these habitats typically support higher abundance and diversity, hydropeaking disturbance carries a disproportionate ecological impact, confirming their particular vulnerability under hydropeaking.

Desiccation risk (M4) addresses a distinct but equally critical stressor for macroinvertebrates, particularly affecting eggs. Elevated risk occurs mainly in wider channel sections with gently sloping banks and bars (Fig. 5g and h), where hydropeaking induces pronounced fluctuations in wetted area (Vanzo et al. 2016; Bätz et al. 2025). These areas frequently experience “slow” and “slow to intermediate” habitats and are commonly selected as oviposition sites, as many aquatic insects attach eggs to partially submerged substrates (Miller et al. 2020; Jaulin Gautronneau et al. 2026). Consequently, drying in these patches directly threatens recruitment, as demonstrated by Judes et al. (2023), who found that the abundance of limnophilic macroinvertebrate communities was reduced by factors between 3.5 to 15.3 depending on the hydraulic conditions experienced in the two weeks preceding sampling. Together with our results, this indicates that the desiccation risk metric captures a life-stage-specific bottleneck linked to repeated wetting–drying cycles (sensu Bätz et al. 2023).

Summarizing, the metrics describe complementary, process-oriented facets rather than providing a single measure of patch-scale habitat dynamics.

### Complementarity and Interactions Among Metrics

The interaction analyses show that the four patch-scale metrics capture largely independent, yet ecologically connected, dimensions of habitat dynamics under hydropeaking. Pairwise correlations are generally weak to moderate, indicating that no single metric can adequately represent macroinvertebrate habitat conditions. Instead, habitat dynamics emerge from the interaction of multiple hydraulic stress mechanisms acting simultaneously and often non-linearly at the patch scale (White et al. 2019; Bätz et al. 2025).

For example, habitat probability and drift risk exhibit a negative correlation, yet a substantial proportion of variance remains unexplained. This indicates that patches with low habitat availability are not necessarily those with high drift exposure, reflecting a physical trade-off between marginal areas prone to drying and hydraulically stressed main-channel sections. A similar inverse pattern emerges between drift and desiccation risk for both habitat types, consistent with previous studies (e.g. Vanzo et al. 2016). However, the residual unexplained variance indicates that this trade-off is not universal, as drift risk can range from moderate to high even where desiccation risk is low. Moreover, hydropeaking frequency may affect these risks in contrasting ways, as more frequent pulses can increase recurrent drift exposure while reducing uninterrupted drying periods in some patches. Finally, weak correlations between habitat probability and habitat shifts show that temporal persistence is not simply a function of habitat availability or hydraulic stress. Frequent habitat shifts occur across patches with differing probabilities, confirming limited persistence as a distinct dimension of hydropeaking impact.

The multimetric clustering integrates these complementary dimensions and reveals recurring patch-scale habitat–risk regimes not evident from individual metrics alone. For both habitat types, Cluster 1 combines moderate habitat availability with comparatively low overall risk, representing the most favorable, though not ideal, conditions for macroinvertebrates under hydropeaking. These patches occupy only a small fraction of the reach and are mainly located in shallow near-bank areas, lateral shelters, and hydraulically sheltered zones behind boulders. In contrast, clusters characterized by low habitat availability and elevated risk are spatially segregated, with higher drift risk (Cluster 2) concentrated within or near the main channel, and desiccation risk (Cluster 3) occurring along channel margins and gently sloping bars. This spatial organization underscores that hydropeaking constrains habitat dynamics through multiple, co-occurring hydraulic stress mechanisms that are strongly structured by channel morphology.

Importantly, the spatial expression of these multimetric regimes reveals that hydropeaking impacts are not uniformly distributed across the channel. Instead, the interaction of hydraulic stress mechanisms gives rise to spatially differentiated responses, whereby different patches experience distinct combinations and intensities of stress. This aligns with previous studies demonstrating that ecological responses to hydropeaking are inherently patchy and conditioned by local hydraulic and morphological context (Shea and Peterson 2007; Bätz et al. 2025). As for instance shown by Tonolla et al. (2023), the combined effects of drift, desiccation, and temporal habitat persistence interfere with essential patch-scale functions such as feeding, dispersal, and reproduction, ultimately resulting in reduced macrozoobenthic biomass and abundance over time. Such disruptions can further induce shifts in community composition (e.g. Ruhi et al. 2018) and propagate through the food web (e.g. Holzapfel et al. 2017). By explicitly capturing these interacting dimensions, the proposed multimetric framework provides a more ecologically meaningful characterization of hydropeaking impacts than single-metric approaches.

### Metrics’ Interactions Response to Structural Complexity

The spatial organization of metric combinations observed in this study reflects the interaction between hydropeaking and river morphology, with structural complexity differing systematically among clusters and acting as a key mediator. While hydropeaking defines the temporal forcing, local structural complexity determines how this forcing is translated into patch-scale habitat conditions and ecological stress (Pringle et al. 1988; Townsend 1989; Palmer and Poff 1997). This is consistent with the concept of a dynamic habitat mosaic, in which local hydraulic stress varies among patches depending on channel structure and discharge variability (Stanford et al. 2005; White et al. 2019; Bätz et al. 2025).

Patches associated with poor habitat condition and moderate to elevated drift risk (Cluster 2) are predominantly located in hydraulically constrained sections with low structural complexity, such as narrow main-channel areas. In these settings, limited bed-level variability amplifies hydraulic stress during discharge increases, leaving little opportunity for discharge refugia to develop (Vanzo et al. 2016; Bätz et al. 2025).

In contrast, patches associated with poor habitat condition and high desiccation risk (Cluster 3) occur mainly along gently sloping banks and bar surfaces in wider sections, where discharge fluctuations translate into pronounced changes in wetted area rather than extreme current velocities (Vanzo et al. 2016; Bätz et al. 2025). These contrasting spatial patterns explain why drift and desiccation risks tend to be spatially segregated (e.g. Vanzo et al. 2016) and emerge as largely distinct hydraulic stress mechanisms, despite partial overlap at the patch scale.

Patches exhibiting the most stable habitat conditions, though still experiencing non-negligible dynamics, and lowest risk levels (Cluster 1) are consistently associated with the highest levels of structural complexity. These patches have limited spatial extent and typically occur in zones influenced by boulders, gravel bars, and lateral shelters, where topographic variability dampens current velocity peaks while maintaining more persistent wetting (Person et al. 2013; Bätz et al. 2025). Such patches may function as partial hydraulic refugia, buffering macroinvertebrates from both excessive current velocities and prolonged drying (Weber et al. 2013). Comparison with previous patch-scale analyses by Bätz et al. (2025) suggests that morphologically complex river configurations, such as braided channels, can substantially reduce hydropeaking-induced patch-scale habitat dynamics, with approximately 40% lower habitat shifts (M2) compared with channelized morphologies. However, these findings currently relate primarily to hydraulically defined habitat types and should not be generalized directly across organism groups.

Accordingly, structurally complex patches in our study enhance temporal habitat persistence, but do not fully offset exposure to hydropeaking-related stressors, reinforcing the view that repeated discharge alteration imposes persistent constraints on habitat dynamics (Bätz et al. 2025; Hayes et al. 2025).

### Uncertainties and Future Research Outlook

While the proposed metrics provide ecologically meaningful insights into macroinvertebrate habitat dynamics under hydropeaking, several sources of uncertainty remain. Rather than limiting the framework, they reflect the current state of knowledge and highlight priorities for further refinement.

A key ecological uncertainty arises from the use of generalized habitat classifications. Although these classes are designed to represent typical hydraulic niches occupied by a wide range of macroinvertebrate taxa, life stages, and functional groups, they inevitably simplify taxon-specific responses within diverse communities (Tonolla 2023; Friese et al. 2025). For example, the desiccation thresholds used here primarily represent egg vulnerability of the genus *Baetis* whereas resistance to drying varies substantially among taxa, with some species producing egg masses that retain moisture for longer periods (Kennedy et al. 2016; Jaulin Gautronneau et al. 2026).

Habitat classification in this study was primarily based on current velocity, a dominant driver of macroinvertebrate distribution. However, other factors such as water depth, substrate composition, temperature, and interstitial connectivity also influence habitat suitability and contribute to broader habitat heterogeneity not captured by the topographic structural-complexity measure used here (Béjar et al. 2020). In particular, substrate clogging and sediment deficits following regulation can reduce interstitial refugia availability and alter food resources, thereby modifying drift and recolonization processes (Dubuis and De Cesare 2023). In addition, horizontal connectivity across the habitat mosaic may play an important role. The current metrics primarily assume limited mobility of macroinvertebrates within fixed patches, whereas more mobile taxa may respond to the spatial configuration and temporal dynamics of the habitat mosaic rather than to local patch conditions alone (White et al. 2019; Béjar et al. 2020). Incorporating complementary descriptors of habitat connectivity and mosaic dynamics, as proposed by Bätz et al. (2025), would extend the framework’s applicability.

Uncertainty also remains in the definition and application of ecological thresholds used to quantify hydropeaking-related stressors. While drift-risk thresholds for “slow” habitats were derived from experimental evidence (Tonolla et al. 2019; Schülting et al. 2023), those for “slow to intermediate” habitats were proportionally extrapolated. Experimental results indicate, however, that drift responses may not scale proportionally across habitat types, depending on community composition and hydraulic context (Friese et al. 2025). Moreover, threshold-based metrics do not fully capture cumulative effects of repeated hydropeaking events. For instance, the first pulse often induces the strongest drift response, while subsequent pulses primarily alter drift composition (Bruno et al. 2016). Similar limitations apply to desiccation-related thresholds. The ecological consequences of repeated dewatering for eggs are only just beginning to be quantified (Jaulin Gautronneau et al. 2026). In addition, desiccation thresholds likely vary with environmental conditions such as temperature, humidity, season, substrate characteristics, and oviposition strategy, which are currently not represented in the metric. Moreover, larval and nymphal stranding during rapid drawdown represent a distinct hydraulic stress mechanism (Tonolla et al. 2023) that is not addressed by the metrics. Together, these limitations highlight cumulative, sequence-dependent, and life-stage–specific responses as a key challenge for future metric development.

### Implications for River Management

Hydropeaking mitigation strategies have historically been developed with a primary focus on fish, despite the fact that benthic macroinvertebrates experience hydropeaking impacts more directly at the patch-scale and often lack the mobility to avoid rapidly changing hydraulic conditions. Traditionally, ecological hydropower mitigation has focused on hydrological measures, such as the implementation of retention basins for gradual water release, or operational adjustments that modify turbine use (Bruder et al. 2016; Bipa et al. 2024). While such operational measures are often constrained by energy production and safe turbine operation requirements, morphological measures and river restoration increasingly aim to enhance structural complexity, particularly in channelized rivers affected by hydropeaking (Person et al. 2013; Friese et al. 2022).

From a management perspective, the results of this study identify structural complexity as an important, yet inherently limited, lever for improving patch-scale macroinvertebrate habitat conditions in hydropeaking rivers. While hydropeaking remains the dominant driver of habitat dynamics, river morphology and instream measures can locally modulate how this hydrological signal is translated into macroinvertebrate-relevant stressors such as drift, desiccation, and temporal habitat persistence (Person et al. 2013; Friese et al. 2022). Consistent with Bätz et al. (2025), increased structural complexity can substantially dampen hydropeaking-induced habitat dynamics, but it cannot fully compensate for altered flow regimes. This distinction is crucial, as it underscores that morphological interventions can reduce exposure to stressors but cannot eliminate hydropeaking impacts altogether. Moreover, the morphological buffering effect may be constrained by additional factors such as substrate deficits and fine sediment clogging following regulation (Dubuis and De Cesare 2023).

The metrics developed here provide a diagnostic tool to evaluate where and how interventions alter patch-scale habitat dynamics (Bätz et al. 2023, 2025). By jointly addressing habitat availability, habitat shifts, drift risk, and desiccation risk, managers can identify critical patches where hydropeaking directly constrains macroinvertebrate survival, recruitment, or colonization potential, and where mitigation is therefore likely to be most effective. Importantly, the framework allows practitioners to distinguish whether interventions reduce drift exposure, limit desiccation, or increase temporal persistence. This supports a process-based river management approach that explicitly accounts for the patchy ecological expression of hydropeaking impacts, rather than relying solely on aggregate impact–discharge relationships (Vanzo et al. 2016; Tonolla et al. 2023; Bätz et al. 2025).

## Conclusion

This study addresses a key gap in hydropeaking impact quantification by explicitly targeting the hydraulic stress mechanisms that shape macroinvertebrate habitat dynamics at the patch scale over a series of frequent hydropeaking events. Our four complementary, spatially explicit and temporally integrated patch-scale metrics capture distinct yet ecologically meaningful dimensions of habitat dynamics that have not been accessible through traditional static-discharge habitat assessment methods. Habitat probability confirms the scarcity of lentic to weakly lotic habitats under hydropeaking, habitat shifts revealed pervasive low temporal persistence, and the risk metrics explicitly identify areas exposed to drift and desiccation. Together, the metrics show that even the most favorable patches remain highly dynamic, underscoring that hydropeaking imposes repeated disturbance well beyond natural variability. The multimetric analysis reveals recurring habitat–risk regimes that explain the spatially patchy organization of macroinvertebrate-related stressors. Structurally complex areas tend to support more favorable metric combinations; however, structural complexity alone does not eliminate hydropeaking-induced stressors, confirming that altered flow regimes remain the dominant driver of habitat dynamics. Importantly, the set of metrics allows these trade-offs to be considered explicitly by distinguishing whether patches are constrained by individual stressors or by interacting combinations of metrics.

The developed framework, implemented as an open-source Python toolbox, is transferable to other river systems, habitat types, and flow regime scenarios by adjusting ecological thresholds, selected habitats, and hydro-morphological inputs. The metrics provide a diagnostic basis to identify ecologically critical patches and evaluate how changes in flow regime or morphology alter macroinvertebrate-relevant hydraulic stress.

As such, the framework supports more process-based, spatio-temporally explicit, and targeted mitigation strategies in regulated rivers.

## Supplementary information

Supplementary information

## Data Availability

Data is available upon request.

## Code Availability

The Python toolbox developed in this study, including source code, user manual, and an example application dataset, is publicly available at: https://github.com/HaDy-toolbox/HaDy_MZB.
