# Supplementary material for: Macroinvertebrate Habitat Dynamics under Frequent Hydropower-Induced Discharge Fluctuations: Patch-Scale Metrics to Quantify Effects of Hydropeaking and Morphological Complexity
Source: Environ Manage. 2026 Aug 18;76(9):279. doi: 10.1007/s00267-026-02574-2 (PMC13486122; doi:10.1007/s00267-026-02574-2)
Supplement: Supplementary file 1 [file 267_2026_2574_MOESM1_ESM.docx]

# Supplementary information

Macroinvertebrate Habitat Dynamics under Frequent Hydropower-Induced Discharge Fluctuations: Patch-Scale Metrics to Quantify Effects of Hydropeaking and Morphological Complexity

Aude Lecrivain ^a,b^, Giovanni De Cesare ^b^, Christine Weber ^a^, Nico Bätz^a^

^a^ Eawag, Swiss Federal Institute of Aquatic Science and Technology, Surface Waters – Research and Management, Kastanienbaum, Switzerland

^b^ EPFL, Hydraulic constructions platform, Station 18, Lausanne, Switzerland

Corresponding author: [nico.baetz@eawag.ch](mailto:nico.baetz@eawag.ch)

ORCIDs numbes:

Aude Lecrivain <https://orcid.org/0009-0003-7436-9302>

Giovanni De Cesare <https://orcid.org/0000-0002-1117-3180>

Christine Weber <https://orcid.org/0000-0003-1404-2247>

Nico Bätz <https://orcid.org/0000-0003-4809-0650>

**Supplementary information 1** Habitat distribution under representative discharge conditions. Panels **(a–c)** show habitat distributions corresponding to the 10^th^, 50^th^, and 90^th^ percentiles of the discharge time-series. Panel **(d)** shows habitat availability across the 13 simulated discharge conditions, expressed as the percentage of the simulated domain (= submerged area at the 99^th^-percentile discharge). Colors correspond to the habitat types defined in the Methods

**Supplementary information 2** Mean silhouette scores for cluster solutions (k = 2–9), used to determine the optimal number of clusters for the k-means analysis (see Methods)
